# Blood and CSF Biomarker Dynamics in Multiple Sclerosis: Implications for Data Interpretation

**DOI:** 10.1155/2011/823176

**Published:** 2011-04-14

**Authors:** M. J. Eikelenboom, B. M. J. Uitdehaag, A. Petzold

**Affiliations:** ^1^Department of Neurology, MS Center Amsterdam, VU University Medical Center, Amsterdam, De Boelelaan 1117, 1081 HV Amsterdam, The Netherlands; ^2^Department of Neuroinflammation, UCL Institute of Neurology, Queen Square, London WC1N 3BG, UK

## Abstract

*Background*. Disability in multiple sclerosis (MS) is related to neuroaxonal degeneration. A reliable blood biomarker for neuroaxonal degeneration is needed. *Objectives*. To explore the relationship between cerebrospinal fluid (CSF) and serum concentrations of a protein biomarker for neuroaxonal degeneration, the neurofilaments heavy chain (NfH). *Methods*. An exploratory cross-sectional (*n* = 51) and longitudinal (*n* = 34) study on cerebrospinal fluid (CSF) and serum NfH phosphoform levels in patients with MS. The expanded disability status scale (EDSS), CSF, and serum levels of NfH-SMI34 and NfH-SMI35 were quantified at baseline. Disability progression was assessed at 3-year followup. *Results*. At baseline, patients with primary progressive MS (PPMS, EDSS 6) and secondary progressive MS (SPMS, EDSS 6) were more disabled compared to patients with relapsing remitting MS (RRMS, EDSS 2, *P* < .0001). Serum and CSF NfH phosphoform levels were not correlated. Baseline serum levels of the NfH-SMI34 were significantly (*P* < .05) higher in patients with PPMS (2.05 ng/mL) compared to SPMS (0.03 ng/mL) and RRMS (1.56 ng/mL). In SPMS higher serum than CSF NfH-SMI34 levels predicted disability progression from baseline (ΔEDSS 2, *P* < .05). In RRMS higher CSF than serum NfH-SMI35 levels predicted disability progression (ΔEDSS 2, *P* < .05). *Conclusion*. Serum and CSF NfH-SMI34 and NfH-SMI35 levels did not correlate with each other in MS. The quantitative relationship of CSF and serum NfH levels suggests that neuroaxonal degeneration of the central nervous system is the likely cause for disability progression in RRMS. In more severely disabled patients with PP/SPMS, subtle pathology of the peripheral nervous system cannot be excluded as an alternative source for blood NfH levels. Therefore, the interpretation of blood protein biomarker data in diseases of the central nervous system (CNS) should consider the possibility that pathology of the peripheral nervous system (PNS) may influence the results.

## 1. Introduction

In multiple sclerosis, irreversible disability progression is anatomically associated with neuroaxonal degeneration [1–3]. Using cerebral microdialysis, it was shown that as a result of neuroaxonal degeneration, protein biomarkers were released into the extracellular fluid (ECF) [4, 5]. Once released into the ECF of the brain, these brain-specific proteins diffuse into the cerebrospinal fluid (CSF) [6]. A protein biomarker specific for neuroaxonal degeneration are neurofilaments [7, 8]. Of the various neurofilament proteins (Nf), the light (NfL) and heavy (NfH) chains were successfully quantified from the CSF and found to be of prognostic value in patients with MS (reviewed in [7, 8] and newer references [9–12]). The Nf proteins diffuse from the CSF into the blood stream from where different NfH phosphoforms have been quantified by different groups [13–15]. Because of the relative ease of blood sampling compared to a spinal tap, it is highly desirable to have a reliable blood biomarker for neuroaxonal degeneration.

There is a need to better understand the relationship between CSF and serum protein biomarkers for neuroaxonal degeneration. The situation in the CSF is relatively straightforward, because any increase can with a considerable degree of confidence be associated with damage to the brain. Interpretation of serum data is more complex. One potentially confounding issue is that Nf are also expressed in the peripheral nervous system [7].

The relationship between CSF and serum NfH levels in MS is not known. In this study, we hypothesized that the well-recognized neuroaxonal degeneration of the central nervous system would lead to a higher concentration in the CSF compared to the serum. To test this hypothesis, we quantified NfH heavy chain phosphoforms from the serum and CSF in a cohort of MS patients we have been published on before [16].

## 2. Methods

### 2.1. Patients

This study was approved by the local ethics committee, and informed written consent was obtained from the patients. All patients with MS were from a previously published Dutch cohort [17] and were classified into having relapsing remitting MS (RRMS, *n* = 21), secondary progressive MS (SPMS, *n* = 22), or primary progressive MS (PPMS, *n* = 9) according to published criteria [18]. Blood and CSF samples were taken at the same time. Matched aliquots of CSF and serum samples were coded and stored in polypropylene tubes as described [19].

### 2.2. Clinical Assessment

Disability was recorded on the expanded disability status scale score (EDSS) [20]. Progression of disability was calculated over the 3-year interval as ΔEDSS = followup EDSS − baseline-EDSS. Significant disability progression was defined as worsening on the EDSS scale by at least 1 point for an EDSS < 5.5 or at least 0.5 point for an EDSS ≥ 5.5.

### 2.3. Neurofilament Analysis

CSF and serum Nf levels were measured using a sensitive sandwich ELISA which allows to quantify various NfH phosphoforms by exchanging the capturing monoclonal antibodies [21]. This ELISA gives the best analytical performance for the monoclonal antibodies SMI34 and SMI35 (originally from Sternberger Monoclonals, now sold through Covance). Adhering to a previously proposed nomenclature NfH captured by SMI34 is indicated as NfH^SMI34^ and NfH captured by SMI35 as NfH^SMI35^. The precise binding epitopes of these antibodies are not known. Binding of SMI34 is phosphate dependent which is correlated with but not identical to the degree of NfH phosphorylation. SMI35 binds more specifically to phosphorylated NfH. Nonmeasurable samples were reported as 0 ng/mL.

### 2.4. Data Analysis

Because of non-Gaussian data distribution the median and interquartile range (IQR) are shown. Nonparametric statistics were used for comparison throughout. We used general linear models for comparison of three variables. The concentration of NfH was compared between CSF and serum for each patient individually. If the concentration of NfH was higher in the CSF compared to the matched serum sample, this was indicated as “C > S”, otherwise as “S ≥ C”. The relationship between higher CSF or serum NfH levels with clinical progression on the EDSS was analyzed using the Kruskall-Wallis test. Correlation analyzes were performed using Spearman's R. The Bonferroni method was used to correct for multiple correlations. All statistical analyzes were performed in SAS (version 9.1).

## 3. Results

### 3.1. Baseline

The demographic data of the MS patients is summarized in Table 1. At baseline, the groups differed in their demographic data for age (*F*_2,48_ = 4.87, *P* < .05), EDSS (*F*_2,48_ = 19,26, *P* < .0001), and disease duration (*F*_2,48_ = 7.33, *P* < .01). None of the biomarkers was correlated with age, age at onset, disease duration, or the EDSS in the MS groups (data not shown).

The only biomarker distinguishing the MS groups were serum NfH^SMI34^ levels (*F*_2,48_ = 3.63, *P* < .05). Significance was missed for CSF NfH^SMI34^ (*P* = .62), CSF NfH^SMI35^ (*P* = .59), and serum NfH^SMI35^ (*P* = .83).

There was no correlation between either the CSF and serum NfH^SMI35^ or NfH^SMI34^ concentration in any of the MS groups (data not shown). The post hoc analysis showed that serum NfH^SMI34^ levels were higher in PPMS patients compared to SPMS patients (*P* = .0128).

The concentration for NfH^SMI35^ was higher in the CSF compared to the serum in 8/9 (89%) of PPMS, 18/22 (82%) of SPMS, and 14/20 (70%) of RRMS patients. Surprisingly, for NfH^SMI34^ the CSF concentration was higher in 2/9 (22%) of PPMS, 11/22 (50%) of SPMS, and 5/20 (25%) of RRMS patients.

### 3.2. 3-Year Followup

The dropout rate for the followup clinical assessment was 4/9 (44%) for PPMS, 4/22 (18%) for SPMS, and 9/20 (45%) for RRMS patients.

At 3-year followup most of the MS patients had progressed clinically on the EDSS scale (Table 2). At followup, there was a difference between the MS groups for the EDSS (*F*_2,31_ = 9.63, *P* < .001) and also for the individual progression on the EDSS (*F*_2,31_ = 3.69, *P* < .05).

Tables 3–5 summarizes the demographic data of the MS patients according to their individual CSF and serum NfH^SMI35^ and NfH^SMI34^ levels.

The low number of patients with PPMS at followup (*n* = 5) precluded any meaningful statistical analyzes. Disability progression (ΔEDSS) appeared to be associated with higher CSF than serum levels for NfH^SMI35^ and higher serum than CSF levels for NfH^SMI34^ (Table 3).

In patients with SPMS, NfH^SMI34^ predicted disability progression (ΔEDSS) from baseline if higher in the serum compared to the CSF (*P* = .0358, Table 4, Figure 2).

**Figure 1:**
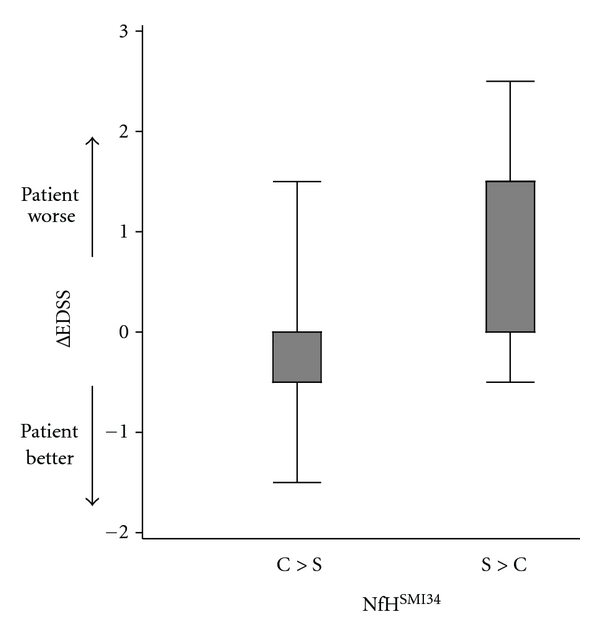
Disability progression in patients with RRMS is associated with higher CSF NfH^SMI35^ levels compared to the corresponding serum concentrations (*P* = .0298) likely indicating neuroaxonal degeneration of the central nervous system. The median (thick horizontal bar), IQR (boxes), and range (whiskers) are shown.

In patients with RRMS, NfH^SMI35^ predicted disability progression (ΔEDSS) from baseline if higher in the CSF compared to the serum (*P* = .0298, Table 5, Figure 1).

## 4. Discussion

The main finding at baseline was that the concentration of NfH phosphoforms was not correlated between matched CSF and serum samples. Unexpectedly, a proportion of patients with MS had higher concentration of NfH phosphoforms in the serum compared to the CSF which was considerable for NfH^SMI34^ (50–88%). Furthermore the absolute concentration of serum NfH^SMI34^ was highest in patients with PPMS (Table 1). This is consistent with the notion that neurodegeneration may be more severe and predominating over inflammation in PPMS [22].

At baseline, there were also demographic differences between patients with RRMS, SPMS and PPMS. Patients with PPMS, and SPMS tended to be older and have a longer disease duration compared to patients with RRMS. These demographic differences did not appear to be related to CSF or blood NfH phosphoform levels, because no correlations were found. This is consistent with other NfH studies on CSF [23] and blood samples [13, 24] based on the same ELISA technique. The present study is underpowered to reveal weak correlations which remain statistical possible. Examining a larger cohort with aid of a newer and more sensitive ECL-based technique compared to our ELISA, age was found to correlated with NfH^SMI35^ levels [12].

Another weakness of this study was the high dropout of patients from baseline to followup. This was likely due to the requirement of a second spinal tap from this community rather than hospital-based cohort of MS patients [16, 25].

At 3-year followup, most patients had progressed on the EDSS, but some did improve (Table 2). Sustained progression on the EDSS was highest for patients with RRMS. Consistent with previous data on this [16] and other cohorts [9–12] of patients with MS, high CSF NfH^SMI35^ levels were of prognostic value (Figure 1). Because in these patients the concentration of CSF NfH^SMI35^ levels was higher compared to the matched serum (Table 5), it is suggested that the NfH^SMI35^ measured was of central origin.

**Figure 2:**
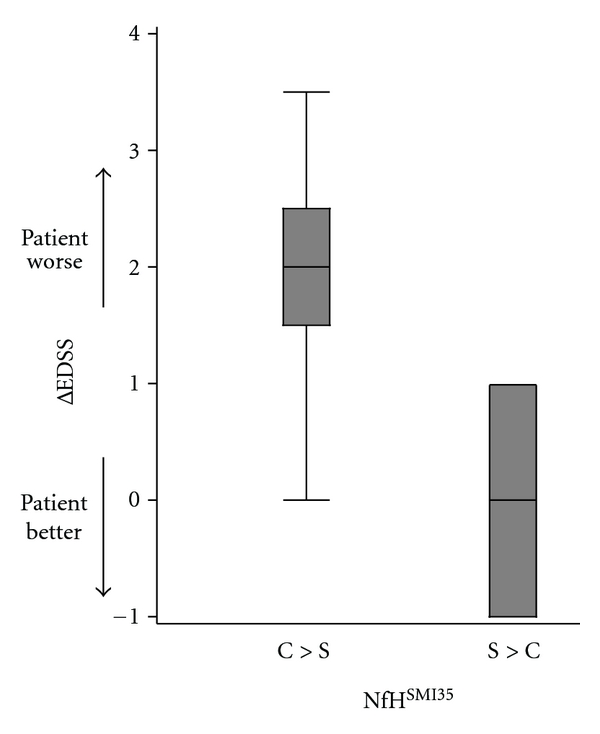
Disability progression in patients with SPMS is associated with higher serum NfH^SMI34^ levels compared to CSF data (*P* = .0358) suggesting that some degree of damage to the peripheral nervous system may exist in these patients.

The followup data on patients with PPMS and SPMS showed a prognostic value for higher concentration of NfH^SMI34^ in the serum compared to the CSF. This was significant for patients with SPMS (Figure 2). Among the number of different explanations, we tentatively suggest that it may be possible that the source for serum NfH^SMI34^ could at least in part originate from the peripheral nervous system. There is some clinical literature supporting this idea. Subtle alterations on routine electrophysiological measurements in patients with MS are found by some [26] but not by others [27]. More sophisticated measurements using nerve excitability measures [28] show changes in the motor nerve recovery cycle, providing indirect evidence for Na^+^/K^+^ ATPase pump dysfunction [29–31], a feature of MS pathology [32]. In addition, teased fibre studies from sural nerve biopsies in MS patients showed more disorganized axonal cytoskeleton (Figure 6 in [33]) similar to what is seen in the brain [34, 35]. In view of this data, we speculate that subtle neuroaxonal degeneration of the peripheral nervous system may be present in more severely disabled PPMS and SPMS patients.

An important limitation of our study is that in absence of specific tests [26–28], there is no direct evidence for damage to the peripheral nervous system. Therefore, this hypothesis will need to be investigated prospectively to be substantiated or defeated. We think this is important in order to ensure that attempts to find a blood-based biomarker for central neuroaxonal degeneration in MS are not contaminated by possible pathology of the peripheral nervous system.

It is noted that the results for NfH^SMI34^ are different to NfH^SMI35^. For NfH^SMI34^, serum levels were frequently higher than CSF levels compared to NfH^SMI35^. Trapp et al. reported changes of NfH phosphorylation, particularly dephosphorylation of demyelinated axons in the MS brain [1]. As MS progresses from RR to SP disease, the burden of altered NfH phosphorylation increases [34–36]. Because NfH^SMI34^ binds to a wider range of NfH phosphoforms than NfH^SMI35^, it may be that serum NfH^SMI34^ levels are more sensitive in detecting axonal damage in MS than serum NfH^SMI35^ levels. This argumentation would be consistent with the finding that a higher concentration of NfH^SMI34^ in the serum compared to the CSF was predictive of disease progression in SPMS. 

Could inflammation-related impairment of the blood brain barrier (BBB) function in MS explain higher blood than CSF NfH levels? We do not think so. Historically, the concept of the BBB originated from the observation that certain compounds did not diffuse freely into the central nervous system (CNS), but they would lead to dramatic symptoms if injected intracerebrally, intraventricularly, or intrathecally [37]. For over a century, research on the BBB has focused on diffusion of compounds *into* the brain. It is now well established that assessment of BBB integrity requires quantification of compounds on both sides of the barrier (reviewed in [38]). In fact, two barriers need to be considered: the morphologically defined BBB and the functionally defined blood CSF barrier (BCB) [6, 38, 39]. Large molecules (e.g., IgM with a molecular weight of 800 kD) can pass the barriers in very small quantities (e.g., IgM serum : IgM CSF = 3000 : 1). Smaller molecules pass through the barriers more easily because of molecular size-dependent diffusion (QAlb = 1 : 200; QIgG = 1 : 500). Starling's principle applies, and an increase of QAlb can be caused by a reduced CSF flow rate without any leakage in the morphological structures [6, 38]. The concept of using a biomarker for parenchymal brain damage (e.g., NfH) for a “BBB leakage” model is incompatible with Starling's principle and the well-established physiology of the BBB/BCB function. Brain-derived proteins in blood can indicate brain damage, as consistently reported by a number of groups, but “leakage” of the BBB/BCB is not a precondition of increased blood concentrations. 

Could the localization of MS lesion formation in the CNS influence whether products of damage are predominantly released into CSF or blood? This certainly is a possibility. The CSF flow dynamics are such that biomarkers released from cortical pathology are likely to diffuse through the cortical arachnoid villi into the blood stream and only a fraction may reach the lumbar CSF [40]. In contrast, pathology of the spinal cord is more likely to be reflected in lumbar CSF. Neurofilaments are one of the few CNS protein biomarkers with a higher lumbar spinal CSF concentration compared to ventricular CSF [41]. The likely reason for this anatomical: there is a rostrocaudal gradient of the parenchymal Nf protein concentration with the lowest concentration in cortical neurons and the highest concentration in spinal cord axons [34, 42, 43]. It could, therefore, be that a small amount of spinal cord damage may mask more extensive cortical damage if investigated from lumbar CSF alone. Conversely, one may hypothesize that blood NfH levels may be better suited for investigating cortical pathology. This hypothesis is tempting, because of the relative ease to obtain serial blood samples as opposed to CSF samples. Precisely for testing, this hypothesis it will be important to ensure that there is no data contamination by pathology of the PNS as tentatively suggested by the present study.

## Figures and Tables

**Table 1:** Patient characteristics at baseline. The median (range) is shown.

Characteristic	PPMS	SPMS	RRMS
Number	9	22	20
Age	52 (46–55)	46 (29–65)	40 (27–55)
Gender (female : male)	6 : 3	11 : 11	11 : 9
Disease duration (years)	16 (6–27)	18 (6–35)	8 (4–13)
EDSS	6 (2–8)	6 (4–7)	2 (1–8)
CSF NfH^SMI34^ (ng/mL)	0.01 (0–0.05)	0.11 (0–0.04)	0.01 (0–0.04)
CSF NfH^SMI35^ (ng/mL)	0.10 (0.01–0.15)	0.04 (0.01–0.24)	0.08 (0–1.39)
Serum NfH^SMI34^ (ng/mL)	2.05 (0–3.08)	0.03 (0.19–2.44)	1.56 (0–2.05)
Serum NfH^SMI35^ (ng/mL)	0 (0–0.49)	0 (0–0.16)	0 (0–0.27)

**Table 2:** Patient characteristics at 3-year followup. The median (range) are shown.

Characteristic	PPMS	SPMS	RRMS
Number	5	18	11
Gender (female : male)	2 : 3	9 : 9	4 : 7
EDSS	7 (4–8)	6 (3–8)	4 (0–5)
ΔEDSS	0 (−1–1.0)	0 (−2–3)	2 (−1–4)

**Table 3:** Primary progressive MS. The patients were classified according to the relationship of CSF and blood NfH levels. If the concentration was higher in the CSF compared to the blood, this was indicated by “C > S” and “S ≥ C” otherwise. The data is presented for each of the two NfH phosphoforms quantified (NfH^*SMI*34^ and NfH^*SMI*35^).

Characteristic	NfH^SMI35^		NfH^SMI34^	
	C > S	S ≥ C	C > S	S ≥ C
Number	4	1	2	3
Age	51 (46–55)	52	48 (46–49)	54 (52–54)
Gender (female : male)	1 : 3	1 : 0	0 : 2	2 : 1
Disease duration (years)	13 (8–20)	17	14 (8–20)	16 (10–17)
EDSS	5 (3–8)	7	7 (7–8)	3 (3–7)
ΔEDSS	1 (−1–1)	0	0 (−1–0)	1 (0–1)

**Table 4:** Secondary progressive MS. The patients were classified according to the relationship of CSF and blood NfH levels. If the concentration was higher in the CSF compared to the blood, this was indicated by “C > S” and “S ≥ C” otherwise. The data is presented for each of the two NfH phosphoforms quantified (NfH^*SMI*34^ and NfH^*SMI*35^). ^⋆^ *P* < .05.

Characteristic	NfH^SMI35^		NfH^SMI34^	
	C > S	S ≥ C	C > S	S ≥ C
Number	15	3	11	7
Age	45 (29–55)	57 (32–65)	49 (32–65)	44 (29–50)
Gender (female : male)	6 : 9	3 : 0	7 : 4	2 : 5
Disease duration (years)	16 (6–28)	22 (15–24)	19 (6–28)	16 (7–22)
EDSS	5 (1–8)	7 (6–8)	6 (3–8)	3 (1–7)
ΔEDSS	0 (−1–3)	0 (−2–0)	0 (−1.5–1.5)	2 (−1–3)^⋆^

**Table 5:** Relapsing remitting MS. The patients were classified according to the relationship of CSF and blood NfH levels. If the concentration was higher in the CSF compared to the blood this was indicated by “C > S” and “S ≥ C” otherwise. The data is presented for each of the two NfH phosphoforms quantified (NfH^*SMI*34^ and NfH^*SMI*35^). ^⋆^ *P* < .05.

Characteristic	NfH^SMI35^		NfH^SMI34^	
	C > S	S ≥ C	C > S	S ≥ C
Number	8	3	5	6
Age	41 (34–53)	38 (29–40)	40 (34–53)	39 (29–48)
Gender (female : male)	4 : 4	0 : 3	2 : 3	2 : 4
Disease duration (years)	10 (4–19)	3 (1–10)	8 (4–13)	9 (1–19)
EDSS	2 (0–4)	2 (1–2)	1 (1–4)	2 (0–2)
ΔEDSS	2 (0–4)^⋆^	0 (−1–1)	2 (0–3)	1 (−1–4)
